# Prognosis stratification and personalized treatment in bladder cancer through a robust immune gene pair‐based signature

**DOI:** 10.1002/ctm2.453

**Published:** 2021-06-20

**Authors:** Xiaofan Lu, Jialin Meng, Junkai Zhu, Yujie Zhou, Liyun Jiang, Yang Wang, Weiheng Wen, Chaozhao Liang, Fangrong Yan

**Affiliations:** ^1^ State Key Laboratory of Natural Medicines Research Center of Biostatistics and Computational Pharmacy China Pharmaceutical University Nanjing 211198 P.R. China; ^2^ Department of Urology The First Affiliated Hospital of Anhui Medical University Institute of Urology Anhui Province Key Laboratory of Genitourinary Diseases Anhui Medical University Hefei 230022 P.R. China; ^3^ Division of Gastroenterology and Hepatology Key Laboratory of Gastroenterology and Hepatology Ministry of Health, Renji Hospital, School of Medicine Shanghai Jiao Tong University Shanghai Institute of Digestive Disease Shanghai 200001 P.R. China; ^4^ Department of Biostatistics The University of Texas MD Anderson Cancer Center Houston Texas 77030 USA; ^5^ Department of Radiology The Affiliated Nanjing Drum Tower Hospital of Nanjing University Medical School Nanjing 210008 P.R. China; ^6^ Department of Endocrinology Zhujiang Hospital of Southern Medical University Guangzhou 510280 P.R. China

To the Editor:

Numerous prognostic signatures to bladder cancer (BCa) have been reported, but many of them limited to either nonmuscle‐invasive (NMIBC) or muscle‐invasive BCa (MIBC), and inherent technical biases across platforms impeded clinical application. As cancer immunity plays a critical role in tumor progression,^1^ we collected 1235 BCa patients from nine independent cohorts across different platforms (Tables S1 and S2) and developed a prognostic signature based on 29 immune‐related gene pairs (IRGPs; Table S3). The entire workflow is depicted in Figure S1, and technical details listed in Supporting Materials and Methods.

We calculated an index (IRGPI, https://github.com/xlucpu/BCaller), which acts as an independent prognostic factor after adjusting other clinicopathological features in metatraining, metatesting, validation 1 and 2 datasets (HR range: 1.55–3.21, all *p *< .05; Figure 1A–E, Figure S2, Tables S4 and S5), and remained highly prognostic for both NMIBCs and MIBCs (Figure 1E). Using dataset‐specific median cutoff, IRGPI stratified patients into low‐ (LRisk) and high‐risk (HRisk) groups (HR range: 2.30–6.13, all *p *< .001; Figure 1F–I) with significant restricted mean survival time ratio (RMS range: 0.42–0.71, all *p *≤ .001; Table S6). Using the cross‐platform cutoff of 1.195, HRisk groups consistently showed poorer outcome than matched LRisk groups (all *p *< .001; Figure S3).

**FIGURE 1:**
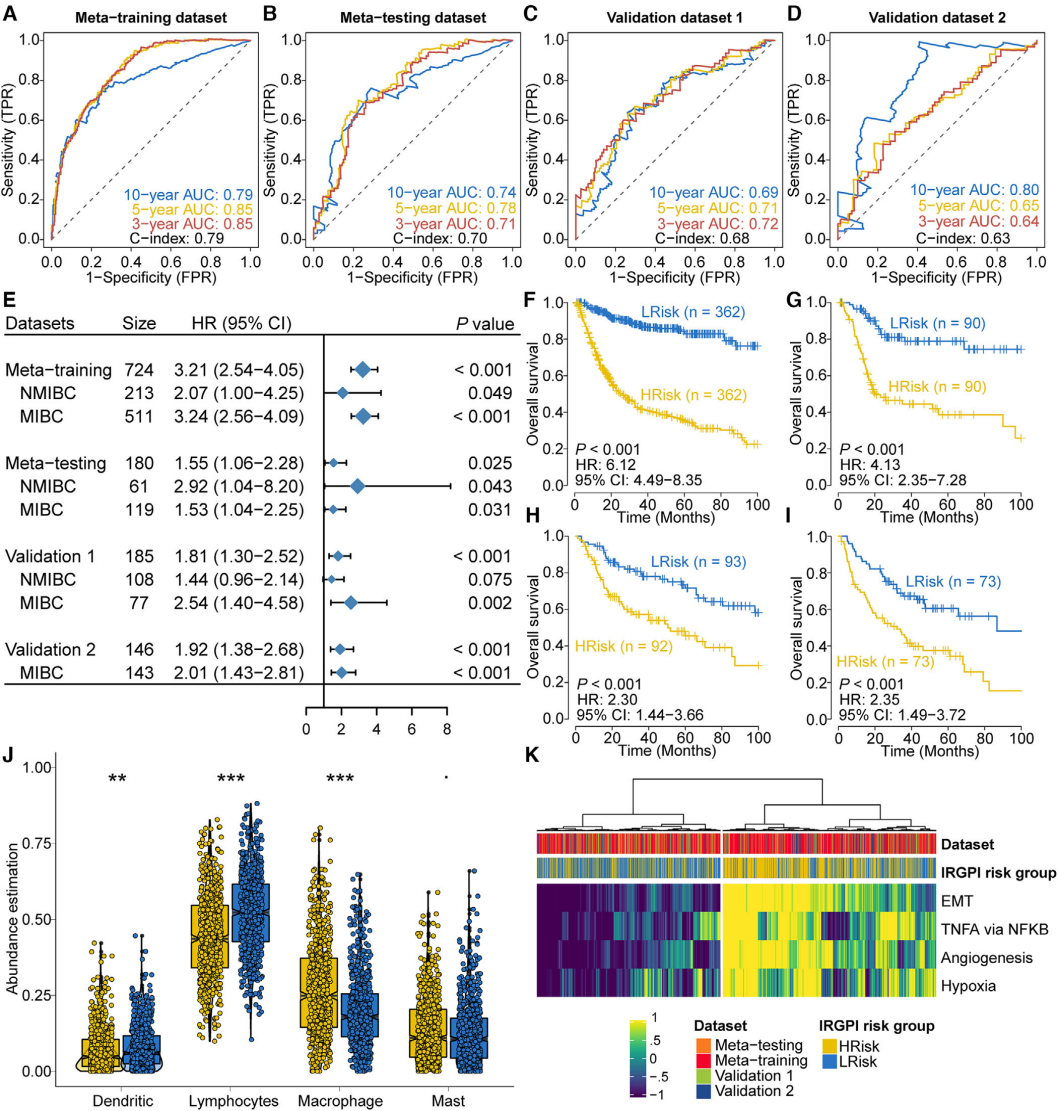
Prognostic value and biological dysfunction of IRGPI. Time‐dependent ROC curve analysis at 3‐, 5‐, and 10‐year survival for four different datasets are shown in (A) to (D), respectively. (E) Forest plot showing the sample size, hazard ratio in multivariate Cox proportional hazards regression after adjusting for major clinicopathological features and the corresponding *p*‐value. Differentiate overall survival probability for patients stratified by IRGPI (HRisk and LRisk groups) was represented by Kaplan–Meier curve from (F) to (I) for metatraining, metatesting, and two validation datasets, respectively. (J) Distribution of four aggregated immune cell types between cohort‐specific HRisk (yellow) and LRisk (blue) groups. (K) Heatmap showing activation of poor survival and immune suppression‐related pathways, including tumor necrosis factor‐α (TNFA), epithelial–mesenchymal transition (EMT), angiogenesis, and hypoxia. *p* < .1, *p < .05, **p < .01, ***p < .001

Functional analyses revealed immune cell‐related pathways were highly enriched for genes within IRGPs (Figure S4A); LRisk showed a higher abundance of lymphocytes and dendritic cells, whereas HRisk enriched macrophages (Figure 1J). Additionally, poor outcome and immune suppression‐related pathways were significantly activated in HRisk groups (Figure 1K, Figure S4B–E), which is concordant with previous literatures.^2^, ^3^, ^4^

We then investigated the genomic variation and molecular subtype between risk groups. We found HRisk group harbored significantly more *TP53* mutations and less *FGFR3* mutations than LRisk (*TP53*: 55.1% vs. 44.0%, *p *= .043; *FGFR3*: 7.5% vs. 18.7%, *p *= .001; Figure 2A), which is consistent with another two cohorts (Figure 2B,C). In 19 BCa cell lines, HRisk cell lines also have two‐fold increased *TP53* mutations than LRisk (*p *= .033). Next, cohort‐specific HRisk groups were more likely to be basal/squamous subtype, whereas most samples within LRisk were predicted as luminal‐papillary (*p *< .05 for eight cohorts; Figure 2D, Figure S5). Given that the interaction of mutations and molecular subtypes and their effects on BCa prognosis have been extensively studied, IRGPI remained an independent prognostic factor after adjusting these prognosis‐associated features (Figure 2E).

**FIGURE 2:**
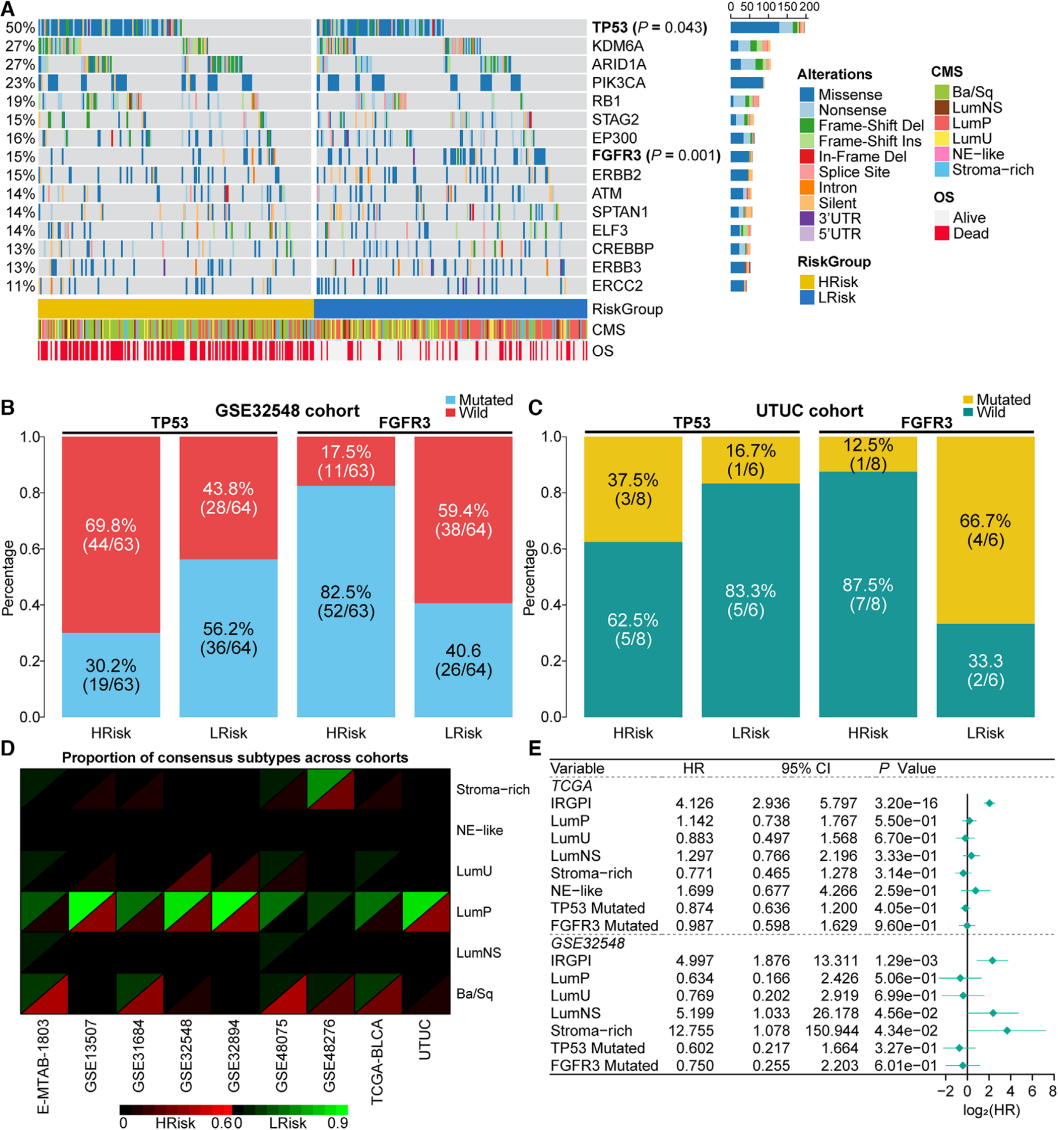
IRGPI was tightly associated with genomic alterations and molecular subtypes of bladder cancers. (A) OncoPrint showing mutational landscape of 15 bladder cancer drivers. (B and C) Barplots showing the similar distribution of *TP53* and *FGFR3* mutations in different risk groups in GSE32548 cohort (*TP53*: 69.8% vs. 43.8%, *p *= .004; *FGFR3*: 17.5% vs. 59.4%, *p <* .001) and UTUC cohort (*TP53*: 16.7% vs. 37.5%, *p *> .05; *FGFR3*: 66.7% vs. 12.5%, *p *= .091), respectively. (D) The consensus subtype frequency of cohort‐specific risk groups across nine cohorts. (E) Forest plot revealed that IRGPI was an independent prognostic factor after adjusting molecular subtypes, *TP53* and *FGFR3* mutations

From a therapeutic standpoint, eight out of nine (88.9%) LRisk groups were highly sensitive to methotrexate (all *p *< .05), while seven (77.8%) HRisk groups were susceptible to paclitaxel (all *p *< .05; Figure 3A, Figure S6), which was validated using 19 BCa cell lines (Figure 3B,C) and consistent with previous study.^5^ Activation of *PI3K‐AKT* signaling might be associated with potential cisplatin chemoresistance in *FGFR3*‐mutant LRisk (Figure S7A) according to the literature.^6^ In another paired BCa cell lines, nine out of 12 samples treated with mitomycin‐C had lower IRGPI than the matched control (*p *= .029; Figure 3D). Additionally, the highly activated complex I/mitochondrial complex in LRisk group may converge to low recurrence rate of mitomycin‐C chemotherapy (Figure S7B).^7^ Since Food and Drug Administration has approved several immune checkpoint blockades for treating BCa, we then applied TIDE (Tumor Immune Dysfunction and Exclusion) algorithm to TCGA‐BLCA and found LRisk group had a significantly higher likelihood of responding to programmed cell death protein 1 antibodies (anti‐PD1) or cytotoxic T‐lymphocyte‐associated protein 4 antibodies (anti‐CTLA4) than HRisk (51% vs. 17.7%, *p *< .001; Figure 3E). We also calculated IRGPI for 298 BCa patients who were treated with programmed death‐ligand 1 antibodies (anti‐PDL1); HRisk patients tended to show an unfavorable long‐term survival after 6 months of treatment (chi‐square [quadratic test] *p *= .07; Figure S8), which may indicate potential resistance to anti‐PDL1 agents. Additionally, we stratified 16 patients with prostate cancer who received anti‐CTLA4 therapy; a remarkably higher number of patients in LRisk group (50%) responded to anti‐CTLA4 treatment than HRisk (12.5%) (Figure 3F). Notably, HRisk group in TCGA‐BLCA showed significantly higher enrichment for immune suppression‐related signatures (all *p *< .001; Figure 3G), which is consistent with our previous study that identified an immunotherapy‐insensitive immune‐exhausted BCa subtype.^8^

**FIGURE 3:**
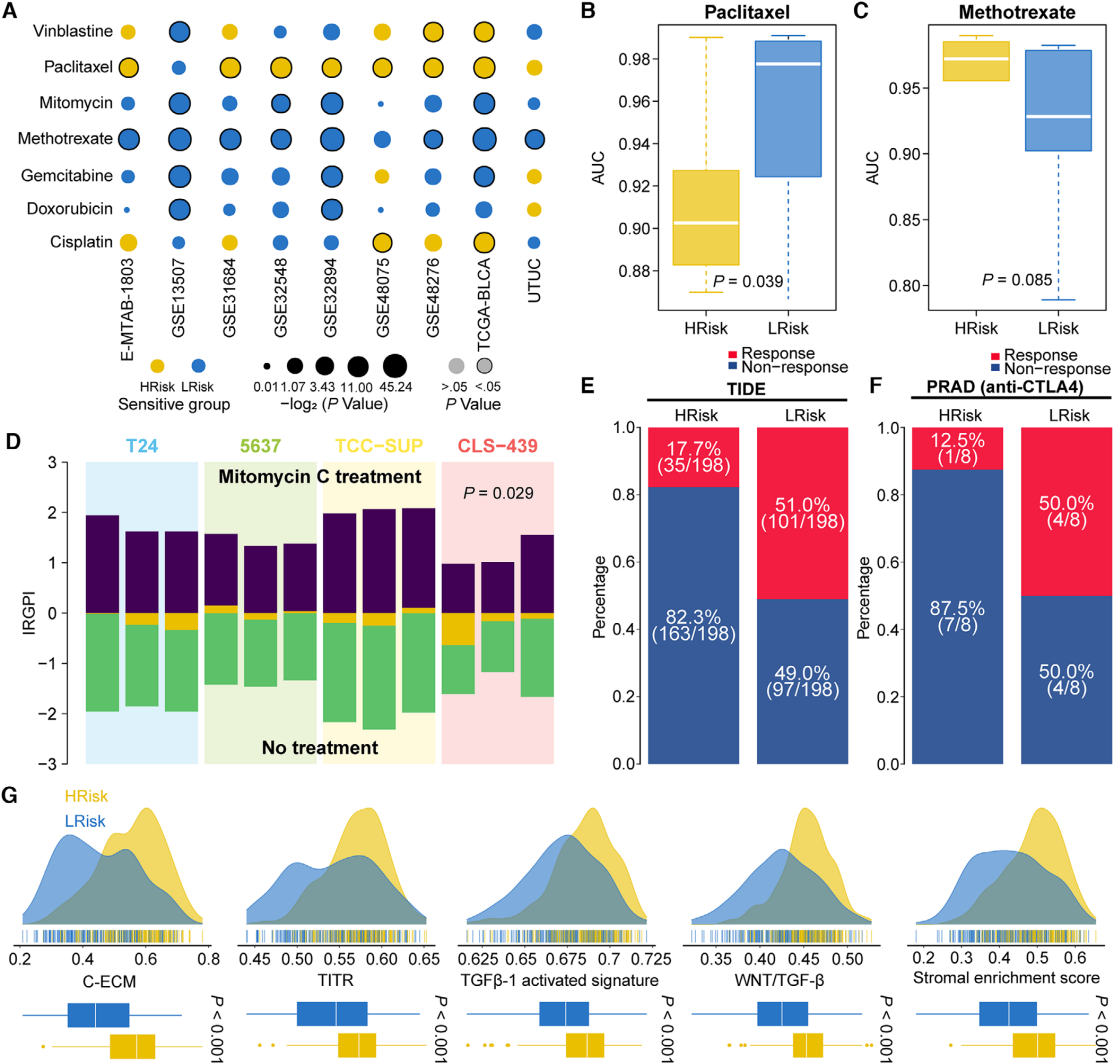
Differentiated response to chemotherapy and immunotherapy between patients stratified by IRGPI. (A) Bubble plot showing the drug sensitivity of HRisk and LRisk groups for seven commonly used chemotherapeutic drugs across nine cohorts. (B and C) Boxplot showing the distribution of sensitivity for paclitaxel and methotrexate between two risk groups stratified by IRGPI in 19 human bladder cancer cell lines. (D) Barplot showing the cell line receiving treatment of mitomycin‐C had lower IRGPI than that of the matched control cell line. If the no‐treatment sample has a higher IRGPI than treatment sample, the difference yellow bar will be located below the *x*‐axis, otherwise the yellow bar will be located above the *x*‐axis. (E and F) Barplots revealed that LRisk group in TCGA‐BLCA cohort might be more sensitive to immune checkpoint blockade; such finding was also consistent with another prostate cancer cohort in which patients who could respond to anti‐CTLA4 had lower IRGPI. (G) HRisk group presented with significant activation of immune‐suppression signatures, including cancer‐associated extracellular matrix (C‐ECM), tumor‐infiltrating Tregs (TITR), transforming growth factor beta 1 (TGF‐β1), Wnt/TGF‐β and stromal signatures as compared to LRisk group in TCGA‐BLCA cohort

To demonstrate the efficiency of IRGPI, we compared it with other prognostic signatures. We first compared IRGPI with two signatures for all‐stage BCa. We demonstrated IRGPI outperformed Cao et al.^9^ signature due to a significantly higher *C*‐index (0.73 vs. 0.63, *p *< .001; Figure 4A, Table S7); IRGPI‐determined risk groups showed a superior survival estimation than using Mo et al.^10^ proposed basal/differentiated subtypes (*C*‐index: 0.75 vs. 0.58; *p *< .001; Figure 4B,C). We then compared IRGPI with four stage‐specific signatures. In NMIBC, increased risk scores calculated by both Heijden et al. and Dyrskjøt et al. signatures significantly associated with poor outcome and were comparable with IRGPI. Comparable or better prediction efficiency was revealed when comparing IRGPI with two MIBC‐based signatures in TCGA‐BLCA cohort, while comparable or inferior performance was achieved in GSE13507‐MIBC cohort (Table S8). To test the robustness of IRGPI, we randomly resampled 80% cases from the entire combined dataset, the NMIBC and the MIBC dataset 10,000 times; all *p‐*values passed the .05 threshold in all‐stage BCa and MIBC datasets, and only 2.65% failed in NMIBC dataset. A satisfactory mean *C*‐index with a relatively low standard deviation also indicated stable predictive power of IRGPI (Figure 4D). Same random resampling of all‐stage BCa, NMIBC, and two cohorts of MIBC was utilized to assess the accuracy and stability of prognostic estimation for other signatures (Figure 4E), indicating the capability of stable and accurate survival prediction by IRGPI‐based models. We further demonstrated IRGPI outperformed other four immune gene‐based prognostic signatures that were trained on TCGA‐BLCA cohort because IRGPI showed the highest prediction performance for 3‐, 5‐, and 10‐year survival (Figure 4F).

**FIGURE 4:**
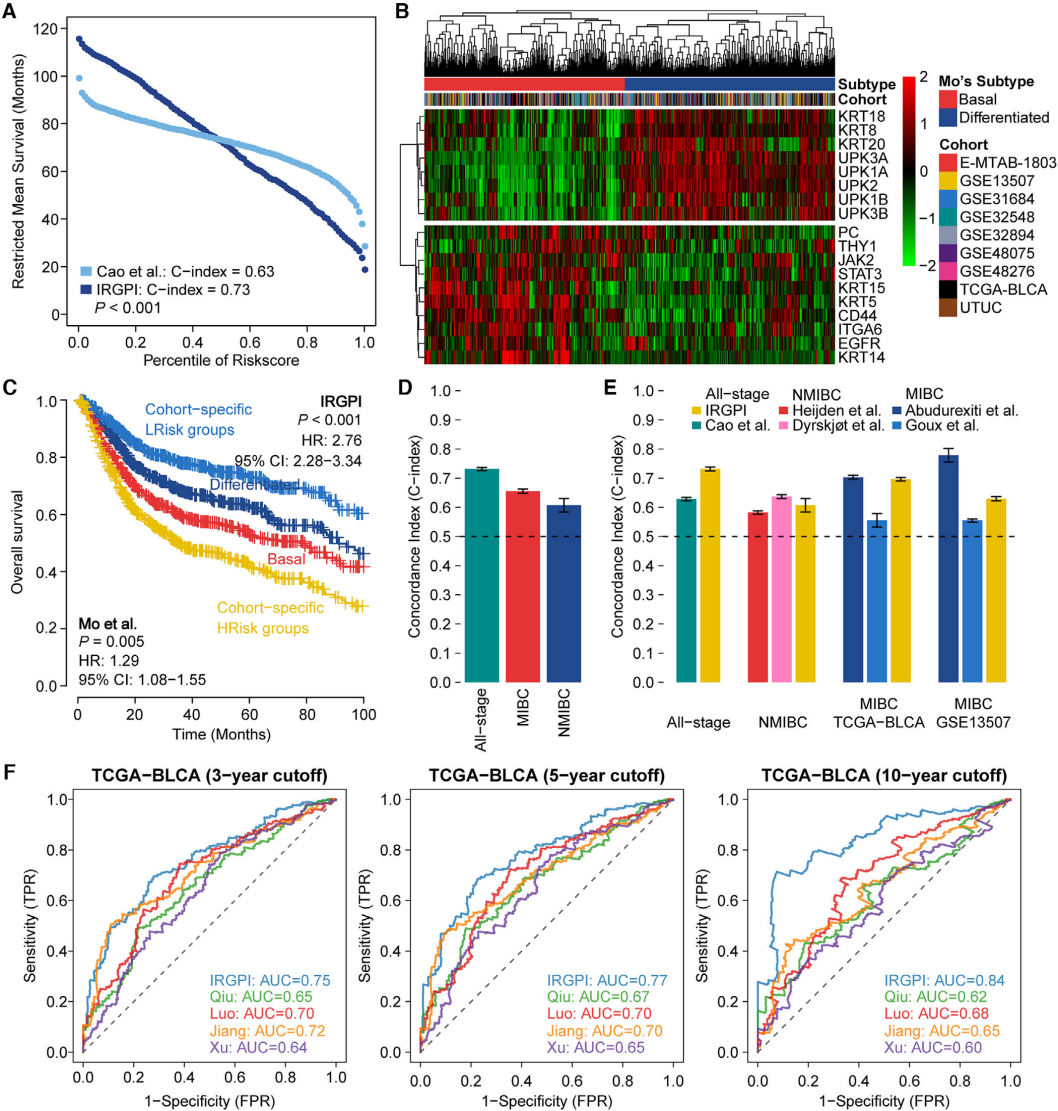
Comparison of IRGPI with other existing prognostic signatures. (A) RMS curve delineating the RMS time of corresponding IRGPI and Cao's risk score. The RMS curve shows a larger slope for IRGPI, demonstrating a better survival estimation as compared to Cao et al. signature. (B) Heatmap showing the distinct expression pattern of Mo's 18‐gene tumor differentiation signature in the entire 1235 BCa samples and two subtypes (i.e., basal and differentiated) are revealed by hierarchical clustering. (C) Kaplan–Meier survival curve showing two pairwise survival curves; that is, IRGPI‐based HRisk and LRisk groups, along with Mo's signature‐based basal and differentiated subtypes. Log‐rank test *p*‐values for survival probability and HR (95% CI) estimated from univariate Cox regressions are also provided for each combination. (D) Average *C*‐index (standard deviation) of IRGPI in all‐stage BCa (0.73 ± 0.006), MIBC‐specific (0.66 ± 0.007) and NMIC‐specific (0.61 ± 0.023) patients. (E) Comparison of *C*‐index between IRGPI and other existing signatures for all‐stage BCa and NMIBC‐specific patients, and MIBC‐specific patients in TCGA‐BLCA and GSE13507 cohorts only, which is represented with mean ± standard deviation. A list of *C*‐index is calculated by resampling 80% of the cases in each subgroup for 10,000 times. Horizontal dashed indicates random prediction (*C*‐index = 0.5). The IRGPI‐based models had a higher but stable *C*‐index in all‐stage BCa than with the Cao et al. signature (0.63 ± 0.007). The IRGPI‐based models demonstrated comparable or superior performance to NMIBC‐based signatures (0.58 ± 0.028 for Heijden et al., 0.64 ± 0.026 for Dyrskjøt et al., and 0.61 ± 0.023 for IRGPI) and comparable or better power than MIBC‐based signatures in the TCGA‐BLCA MIBC dataset (0.70 ± 0.011 for Abudurexiti et al., 0.56 ± 0.012 for Goux et al., and 0.70 ± 0.010 for IRGPI), whereas comparable or inferior performance was achieved with the GSE13507‐MIBC dataset (0.78 ± 0.027 for Abudurexiti et al., 0.56 ± 0.031 for Goux et al., and 0.63 ± 0.026 for IRGPI). (F) Four immune gene‐based prognostic signatures trained on TCGA‐BLCA cohort were assessed for performance with IRGPI; the prediction efficiency was compared through AUC calculated by time‐dependent ROC analyses at 3‐, 5‐, and 10‐year survival

We acknowledged limitations. The high missing rate of clinical characteristics may decrease the statistical power in multivariate analysis. Prospective studies are needed to further test IRGPI's clinical utility in the individualized management. In summary, this study highlights the importance of IRGPI that can be used not only to predict survival but also to investigate personalized treatment strategies. Notably, IRGPI serves as a single‐sample survival estimator of BCa and may be readily translated to clinical practice to guide prognosis stratification and personalized treatment.

## CONFLICT OF INTEREST

The authors declare that there is no conflict of interest.

## ETHICS STATEMENT

As the data used in this study are publicly available, no ethical approval is required.

## AUTHOR CONTRIBUTIONS

Conceptualization: Xiaofan Lu and Jialin Meng. Methodology: Xiaofan Lu, Junkai Zhu, and Liyun Jiang. Formal analysis: Xiaofan Lu, Jialin Meng, Junkai Zhu, Yujie Zhou, and Liyun Jiang. Investigation: Xiaofan Lu, Jialin Meng, Junkai Zhu, Yujie Zhou, Yang Wang, and Weiheng Wen. Writing original draft: Xiaofan Lu and Jialin Meng. Visualization: Xiaofan Lu, Jialin Meng, and Yujie Zhou. Funding acquisition: Chaozhao Liang and Fangrong Yan. Supervision: Chaozhao Liang and Fangrong Yan.

## DATA AVAILABILITY STATEMENT

Raw data for this study were generated at corresponding archives. Derived data supporting the findings are available from the corresponding author (Fangrong Yan) upon reasonable request. An R package “*BCaller*” (https://github.com/xlucpu/BCaller) was used to calculate an immune‐related genes pair index (IRGPI) from single‐sample perspective using transcriptome profiles for bladder cancer.

## Supporting information

Supporting Information
