# Supplementary material for: Prognosis stratification and personalized treatment in bladder cancer through a robust immune gene pair‐based signature
Source: Clin Transl Med. 2021 Jun 20;11(6):e453. doi: 10.1002/ctm2.453 (PMC8214857; doi:10.1002/ctm2.453)
Supplement: Supplementary file 1 — Supporting Information [file CTM2-11-e453-s001.docx]

**PROGNOSIS STRATIFICATION AND PERSONALIZED TREATMENT IN BLADDER CANCER THROUGH A ROBUST IMMUNE GENE-PAIR BASED SIGNATURE**

**INDEX OF SUPPLEMENTARY MATERIALS**

**SUPPLEMENTARY MATERIALS AND METHODS……………………………Page 2**

**SUPPLEMENTARY TABLES S1-8 ………………………………………………. Page 9**

**SUPPLEMENTARY FIGURES S1-8 ……………………………………………... Page 15**

**REFERENCES ……………………………………………...…………………..……Page 22**

**MATERIALS AND METHODS**

**Study population and exclusion criteria**

We retrospectively collected gene expression profiles from frozen bladder cancer tumour tissue samples from nine public bladder cancer (BCa) cohorts: seven microarray cohorts with one from ArrayExpress and six from Gene Expression Omnibus (GEO); one RNA-Seq cohort from The Cancer Genome Atlas (TCGA) project of TCGA-BLCA; and a cohort of another 20 upper-tract urothelial carcinoma (UTUC) samples quantified by RNA-Seq published in a previous study [1]. Only patients with available overall follow-up time, overall survival (OS) status and gene expression data were included initially. Of the tumours from these patients, we excluded samples whose OS time was less than one month to enhance the robustness of downstream analyses. The right-censoring was defined as loss to follow-up. No limitation was applied for age, sex, histology type, tumour stage (T-stage), tumour grade, muscle-invasiveness status or neoadjuvant therapies for sample collection. Corresponding clinicopathological information of the remaining eligible 1,235 samples was retrieved from cBioPortal (<http://www.cbioportal.org/datasets>) for the TCGA cohort, GEO (<https://www.ncbi.nlm.nih.gov/geo/>) and ArrayExpress (<https://www.ebi.ac.uk/arrayexpress/>) for other microarray cohorts under the corresponding archives, and BioProject (PRJNA678814) for the UTUC cohort. Somatic mutation data were downloaded from PanCanAtlas and filtered for urothelial bladder carcinoma (BLCA) tumour type in the TCGA cohort. Whole-exome sequencing data for 14 available UTUC samples were also retrieved for mutation calling, as described previously [1]. Information about the platform and corresponding sample size of the nine BCa cohorts are summarized in Table S1.

**Partition of datasets**

Given the completeness of the clinical information of the GSE13507 (n=165) and UTUC (n = 20) cohorts, these two cohorts were combined as independent validation set 1 with a total of 185 samples; due to insufficient information on sex and tumour grade, two GEO cohorts (*i.e.*, GSE48075 and GSE48276) were selected and combined as independent validation dataset 2, comprising a total of 146 BCa samples. The remaining five cohorts, including 904 BCa samples (meta-cohort dataset), were randomized into two subsets based on 5-fold sampling, where the meta-training dataset included four folds of BCa samples (n = 724) and the internal meta-testing dataset included the rest (n = 180). Patients with early-stage BCa were determined as samples with a T-stage of Ta and T1, indicating that these tumours were only in the innermost layer of the bladder lining (Ta) or had started to grow into the connective tissue beneath the bladder lining (T1); these tumours were commonly classified as NMIBC. A total of 385 samples were considered early-stage BCa or NMIBC (three samples from the TCGA-BLCA cohort and one sample from GSE13507 with a confirmed status of muscle invasiveness were discarded). We also curated MIBC samples, including 850 tumour samples derived from the TCGA-BLCA cohort (n = 396), GSE13507 cohort (n = 62), and UTUC cohort (n = 15) with confirmed status, and samples from other cohorts with T-stages of T2, T3 and T4.

**Preprocessing of gene expression profiles**

For raw counts of high-throughput sequencing data retrieved from TCGA-BLCA and UTUC cohorts, Ensembl IDs were transformed to gene symbols by mapping with GENCODE27. The number of fragments per kilobase of nonoverlapping exons per million fragments mapped (FPKM) was computed first and transferred into transcripts per kilobase million (TPM) values, which showed more similarity to those generated from microarray and were more comparable between samples [2]. For the microarray data, the probe ID was annotated to gene symbols according to the corresponding platform’s annotation file. For multiple probes that mapped to one gene, the mean value of expression was considered. To minimize the cross-cohort batch effect, each dataset was logarithmically converted if necessary, and gene-wise location scaling within each cohort was further conducted [3]. After removing genes with consistent expression in each dataset, a total of 12,344 unique genes were shared across all nine cohorts.

**Identification of immune-related gene pairs**

We followed and modified the technical details of building gene-pair signature according to previous literatures [4-8]. To be specific, we collated a list of immune-related genes (IRGs) from the nCounter PanCancer Immune Profiling Panel that contained 770 unique genes closely associated with the human immune response in both solid and liquid cancer types [9]. A total of 677 IRGs measured in all cohorts were selected. The gene expression level in a specific sample underwent pairwise comparison to generate a score for each pair of IRGs (*i.e.*, IRGPs). An IRGP score of 1 was assigned if the former IGR was less than the latter IGR; otherwise, 0 was assigned. Such a gene pair-based approach demonstrates evident merit compared to traditional expression-based methods because the score is computed based entirely on the relative expression level within the same cohort; in this context, normalization of expression data is unnecessary. IRGPs presenting with constant scores in a particular cohort might be attributable to platform-dependent preferential measurement, which can cause biases and may not be reproducible across cohorts, or to biologically preferential transcription, which failed to provide discriminative information for prognosis [10]; thus, IRGPs with constant scores in any cohort were removed.

**Development of a prognostic signature based on immune-related gene pairs**

Prognostic IRGPs were filtered out by using a univariate Cox proportional hazards regression model to assess the association between each IRGP and patient OS in the meta-training dataset. Because a tremendous number of significant prognostic IRGPs were detected (*P* < 0.05), we set a stringent threshold for initial filtration for prognostic IRGPs (Bonferroni-corrected *P* values less than 0.05 and hazard ratio (HR) greater than 1.5 or less than 1/1.5). To minimize the risk of overfitting, we used the “*glmnet*” R package and applied a multivariate Cox proportional hazards regression model with the adaptive least absolute shrinkage and selection operator (adaLASSO), which adds weights to traditional LASSO to counteract the known issue of bias in LASSO estimates [11]. Ten-fold cross-validation was conducted to tune the optimal value of penalty parameter $\lambda$ that gives the minimum partial likelihood deviance. Finally, a set of prognostic IRGPs and their non-zero coefficients were determined to build an IRGP index (IRGPI) for each sample via a linear combination of the selected features, weighted by the corresponding coefficients as follows:

$$S_{IRGPI}=\sum_{i=1}^{n} C_{i}\times B_{i}$$

where $C_{i}$ is the coefficient, $B_{i}$ is the binary score of the IRGP, and $S_{IRGPI}$ is the computed score for the IRGPI. Patients were dichotomized into high-risk (HRisk) and low-risk (LRisk) groups using the dataset-specific median IRGPI as the cut-off. Patients could also be divided into two risk groups according to the cohort-specific median cut-off. An R package “BCaller” (<https://github.com/xlucpu/BCaller>) was offered to calculate an immune-related genes pair index (IRGPI) from single-sample perspective using transcriptome profiles for bladder cancer.

**Validation of IRGPI**

The prognostic value of the IRGPI was evaluated in patients at all stages of BCa in stage-specific groups in meta-training and meta-testing and in two independent validation datasets by univariate analyses. We also assessed the independent prognostic effect of IRGPI by adjusting for other available clinicopathological variables in the multivariate analyses, including age, sex, tumour grade and T stage, if available. Among these variables, IRGPI and age (per ten years) were treated as continuous forms, and the remaining three were coded as binary. Specifically, tumour grades recorded in different cohorts that were defined as “high grade”, “G2” or “G3” were unified as “poorly differentiated”, and “low grade” or “G1” was unified as “well/moderately differentiated”. For the T-stage, which generally included five categories (Ta, T1, T2, T3 and T4), we coded Ta and T1 as early-stage BCa (*i.e.*, NMIBC) and the remaining categories as advanced-stage BCa (*i.e.*, MIBC). The prognostic accuracy of the continuous IRGPI was determined via the concordance index (C-index), which ranges from 0 to 1, with 0.5 indicating random estimation.

**Bioinformatic analyses**

To gain a biological understanding of the IRGPI, we conducted Gene Ontology (GO) and Kyoto Encyclopedia of Genes and Genomes (KEGG) analyses, and gene set enrichment analysis (GSEA) was conducted through the R package “*clusterProfiler*” [12]. Specifically, GSEA was performed based on a pre-ranked gene list sorted by log2FoldChange derived from limma differential expression analysis [13, 14]. We analysed four aggregated immune cell types, namely, total lymphocytes, total dendritic cells (the sum of activated and resting dendritic cell percentages), total macrophages (the sum of M0, M1 and M2 macrophage percentages) and total mast cells (the sum of activated and resting mast cell percentages), according to the literature [15]. Signature of PI3K–AKT pathway was extracted from the literature [16]. Three complex I/mitochondrial complex associated pathways were download from MSigDB, including RESPIRATORY_CHAIN_COMPLEX_I, WONG_MITOCHONDRIA_GENE_MODULE, and KEGG_OXIDATIVE_PHOSPHORYLATION. Enrichment scores for signatures/pathways were calculated by the single sample GSEA approach by using the R package “*GSVA*” [17]. The mutation landscape was analysed by the R package “*maftools*” following the initial removal of 100 FLAGS genes [18]. Cancer driver mutations for BCa were identified according to the literature as having a mutation rate greater than 10% [19]. We used the R package “*consensusMIBC*” to predict individual consensus molecular subtypes (CMSs) for BCa, including basal/squamous, luminal papillary, luminal unstable, luminal non-specified, neuroendocrine-like, and stroma-rich subtypes [20].

**Prediction of chemotherapy and immunotherapy sensitivity**

Based on the largest publicly available pharmacogenomics database (the Genomics of Drug Sensitivity in Cancer [GDSC], <https://www.cancerrxgene.org/>), we employed the R package “*pRRophetic*” to predict the chemotherapeutic sensitivity for each BCa case; the estimated IC_50_ of each case treated with a specific chemotherapy drug was obtained by ridge regression, and the prediction accuracy was measured through 10-fold cross-validation with the GDSC training set [21]. Tumour Immune Dysfunction and Exclusion (TIDE) was used to predict the clinical response to immune checkpoint blockades [22].

Two external cohorts were used to validate chemotherapy sensitivity. First, we downloaded and extracted transcriptome expression, genomic alteration and compound sensitivity data (measured as the area under the dose-response curve—AUC; a lower AUC indicates increased sensitivity to treatment) for 19 human bladder cancer cell lines from the Broad Institute Cancer Cell Line Encyclopedia (CCLE) project [23]. The IRGPI was calculated for each cell line, and the drug sensitivity of each specific compound was compared. Second, we retrieved expression data from the GSE151505 cohort, which included 12 matched samples of bladder cancer cell lines [24]. This cohort contains four types of cell line (*i.e.*, T24, 5637, TCC-SUP and CLS-439) with a total of 24 samples, and half of the matched cell lines received mitomycin C treatment, whereas the other half was treated as a control. For visualization, if the no-treatment sample has a higher IRGPI than treatment sample, the difference bar will be located below the x-axis of the barchart, otherwise the difference bar will be located above the x-axis of the barchart. The IRGPI difference was computed for the matched cell lines, and we tested whether the direction of the difference was related to the treatment.

Two external cohorts were used for immunotherapy. First, we extracted the raw transcriptome count data and clinicopathological and survival information for 298 BCa patients who were from a large phase 2 trial investigating the clinical activity of anti-PD-L1 blockade of atezolizumab in locally advanced and metastatic urothelial carcinoma (mMIBC) using the R package “*IMvigor210CoreBiologies*” [25]. All these patients have complete records of response (*i.e.*, complete response, partial response, stable disease, and progressive disease) to PD-L1 blockade. Raw count data were converted to TPM values, and individual IRGPIs were computed. Given the delated clinical effect of immunotherapy, the association between the risk group and PD-L1 blockade response was reflected by long-term survival analysis after 6-month of treatment. Second, we retrieved raw count data from the literature for 18 patients with prostate cancer who received hormone therapy with castrate serum testosterone, but still demonstrated tumor progression, and then received anti-CTLA4 antibodies (a phase 2 clinical trial of ipilimumab [NCT02113657]) [26]. Similarly, TPM values and individual IRGPIs were calculated. The association between the risk group and response to anti-CTLA4 treatment was further analysed. Patients were stratified according to the median cut-off of IRGPI in both cohorts.

**Existing prognostic signatures for comparison**

To assess the survival classification and prediction ability of the IRGPI, we retrospectively collected ten published BCa prognostic signatures for comparison. Gene aliases from different studies were manually unified through GeneCards ([https://www.genecards.org](https://www.genecards.org/)) to ensure that all genes involved in each signature could be matched in this study. Specifically, Cao *et al*. proposed a seven EMT-related gene signature to predict OS based on a pregenerated formula for risk score calculation [27]. Mo *et al*. reported an 18-gene tumour differentiation signature to stratify BCa samples and revealed two distinct subtypes with significantly different prognoses by hierarchical clustering [28]. To reproduce these two subtypes, we mapped all these genes and performed supervised hierarchical clustering on the z-score transformed expression matrix of the entire 1,235 BCa samples with distance measurement of 1-Pearson correlation coefficient and the “complete” agglomeration method [28]. Two signatures used to improve the prognostic prediction for early-stage or NMIBC were also evaluated, including a 5-gene signature proposed by Heijden *et al*. [29] and a 12-gene signature built by Dyrskjøt *et al*. [30]. We also assessed two prognostic signatures for MIBC, including a 3-gene signature developed by Goux *et al*. [31] and a 12-gene signature constructed by Abudurexiti *et al*. [32] by analysing MIBC samples from the TCGA-BLCA and GSE13507 cohorts. Since these four stage-specific prognostic signatures were identified by multivariate Cox proportional hazards regression, we applied them to either our NMIBC samples or a subset of MIBC samples via multivariate analyses. Only those samples with confirmed muscle invasiveness status in the TCGA-BLCA (n = 396) and GSE13507 (n = 62) cohorts were used to evaluate the MIBC-based signature according to the literature [32]. Risk scores were calculated by multiplying gene expression values by their corresponding coefficients and summing these values. The *P* values of continuous risk scores or dichotomized subtypes in the univariate Cox proportional hazards regression model and the overall C-index were further compared. Since the IRGPI was identified by using immune-related gene pairs, four immune gene-based prognostic signatures trained on TCGA-BLCA cohort were also assessed for performance with the IRGPI [33-36]; the prediction efficiency was compared by using receiver operating characteristic (ROC) analyses for 3-year, 5-year and 10-year survival.

**Statistical analyses**

All statistical analyses were conducted by R4.0.2 using a two-sample Mann-Whitney test for continuous data, which is represented as the mean ± standard deviation (SD), and Fisher’s exact test for categorical data. For survival analyses, a Kaplan-Meier curve was generated for survival rates of patients with difference detection performed with a log-rank test. Cox regression was used to calculate the OS hazard ratios (HRs) and 95% confidence intervals (CIs). Given the delayed clinical effect, treatment effect of immune checkpoint inhibitor was measured by non-proportional hazards statistical approach of long-term survival inference by using R package “*ComparisonSurv*” [37, 38]. A C-index was calculated with the “*survcomp*” R package and compared with the “*compareC*” R package. In addition, the prediction efficiency of the IRGPI for 3-year, 5-year and 10-year survival was examined using ROC analyses by R package “*survivalROC*”. The restricted mean survival (RMS) curve and RMS time ratio were estimated by the “*survival*” and “*survRM2*” R packages. RMS represents the life expectancy at 120 months (10 years) and the performance of risk groups determined by the IRGPI was assessed with reference to the RMS time ratio; the higher the RMS value is, the greater the prognosis difference. For all statistical analyses, a two-tailed *P* value less than 0.05 was considered statistically significant.

**SUPPLEMENTARY TABLES**

**Supplementary Table S1.** Summary of the nine cohorts included in the study.

| **Cohorts** | **Archive** | **Platform** | **Data Type** | **Sample Size** |
| --- | --- | --- | --- | --- |
| TCGA-BLCA | TCGA | Illumina HiSeq 2000 RNA Sequencing | RNA-Seq | 396 |
| E-MTAB-1803 | ArrayExpress | Affymetrix Human Genome U133 Plus 2.0 Array | Microarray | 70 |
| GSE13507 | GEO | Illumina human-6 v2.0 expression beadchip | Microarray | 165 |
| GSE31684 | GEO | Affymetrix Human Genome U133 Plus 2.0 Array | Microarray | 90 |
| GSE32548 | GEO | Illumina HumanHT-12 V3.0 expression beadchip | Microarray | 127 |
| GSE32894 | GEO | Illumina HumanHT-12 V3.0 expression beadchip | Microarray | 221 |
| GSE48075 | GEO | Illumina HumanHT-12 V3.0 expression beadchip | Microarray | 73 |
| GSE48276 | GEO | Illumina HumanHT-12 WG-DASL V4.0 R2 expression beadchip | Microarray | 73 |
| UTUC- PRJNA678814 | BioProject | Illumina HiSeq 2000 RNA Sequencing | RNA-Seq | 20 |

**Supplementary Table S2.** Demographic and clinic characteristic descriptions for bladder cancer patients in different datasets.

| **Characteristics ^a^** | **TCGA-BLCA** | **E-MTAB-1803** | **GSE13507** | **GSE31684** | **GSE32548** | **GSE32894** | **GSE48075** | **GSE48276** | **UTUC** |
| --- | --- | --- | --- | --- | --- | --- | --- | --- | --- |
| Number of samples | 396 | 70 | 165 | 90 | 127 | 221 | 73 | 73 | 20 |
| Median survival time  (month) (95% CI) | 34.0  (27.0-56.3) | 26.0  (19.0-60.0) | 87.1  (66.3-NA) | 51.5  (17.0-90.1) | NA ^b^  (NA-NA) | NA  (NA-NA) | 37.2  (18.7-82.4) | 68.8  (37.5-NA) | 59.8  (49.0-NA) |
| Number of Death (%) | 176 (44.4) | 42 (60.0) | 69 (41.8) | 63 (70.0) | 23 (18.1) | 25 (11.3) | 45 (61.6) | 34 (46.6) | 7 (35.0) |
|  |  |  |  |  |  |  |  |  |  |
| Age (Years) ^c^ | 68.0 ± 10.6 | 67.1 ± 11.2 | 65.2 ± 12.0 | 69.0 ± 10.2 | 69.5 ± 10.6 | 69.4 ± 11.3 | 68.8 ± 10.2 | 65.7 ± 9.9 | 71.6 ± 11.2 |
| Gender |  |  |  |  |  |  |  |  |  |
| Female | 105 | 11 | 30 | 24 | 30 | 60 | - | 14 | 2 |
| Male | 291 | 59 | 135 | 66 | 97 | 161 | - | 59 | 18 |
| Histology type (%) |  |  |  |  |  |  |  |  |  |
| urothelial | 396 | - | - | - | - | - | - | 57 | 20 |
| micropapillary | 0 | - | - | - | - | - | - | 3 | 0 |
| sarcomatoid differentiated | 0 | - | - | - | - | - | - | 1 | 0 |
| squamous differentiated | 0 | - | - | - | - | - | - | 11 | 0 |
| focal glandular | 0 | - | - | - | - | - | - | 1 | 0 |
| Grade |  |  |  |  |  |  |  |  |  |
| Well/moderately differentiated | 18 | 0 | 105 | 6 | 15 | 44 | - | - | 0 |
| Poorly differentiated | 375 | 70 | 60 | 84 | 112 | 175 | - | - | 20 |
| T-stage |  |  |  |  |  |  |  |  |  |
| Ta | 0 | 0 | 24 | 5 | 40 | 109 | 0 | 0 | 2 |
| T1 | 3 | 0 | 80 | 10 | 49 | 61 | 0 | 3 | 3 |
| T2 | 114 | 24 | 31 | 17 | 37 | 43 | 41 | 13 | 5 |
| T3 | 191 | 28 | 19 | 40 | 0 | 7 | 23 | 41 | 7 |
| T4 | 55 | 18 | 11 | 18 | 0 | 1 | 8 | 10 | 3 |
| Muscle-invasiveness ^d^ |  |  |  |  |  |  |  |  |  |
| NMIBC ^e^ | 0 | 0 | 103 | 15 | 89 | 170 | 0 | 3 | 5 |
| MIBC ^f^ | 396 | 70 | 62 | 75 | 37 | 51 | 72 | 64 | 15 |
| ^a^ Sum of frequency numbers may not equal to the total sample size due to missing or unpredictable values | | | | | | | | |  |
| ^b^ Median survival time is incalculable because the mortality at the last follow-up time is less than 50% | | | | | | | | |  |
| ^c^ Age is represented as mean ± standard deviation | | | | | | | | |  |
| ^d^ Cohorts of TCGA-BLCA and GSE13057 record confirmed status of muscle-invasiveness. | | | | | | | | |  |
| ^e^ NMIBC: non-muscle invasive bladder cancer (Ta + T1 or non-muscle invasiveness confirmed). | | | | | | | | |  |
| ^f^ MIBC: muscle invasive bladder cancer (T2 + T3 + T4 or muscle-invasiveness confirmed). | | | | | | | | |  |

**Supplementary Table S3.** Model information about IRGPI.

| **IRGP** | **IRGP1** | **IRGP2** | **AdaLASSO Coefficient** | **HR (95% CI)** | **Adjusted *P*** |
| --- | --- | --- | --- | --- | --- |
| P1 | AMMECR1L | MST1R | -0.2781 | 0.4905 (0.3809-0.6316) | 0.0017 |
| P2 | BIRC5 | ST6GAL1 | -0.2405 | 0.2571 (0.1635-0.4043) | 0.0002 |
| P3 | G6PD | SYT17 | -0.2315 | 0.1932 (0.1325-0.2815) | <0.0001 |
| P4 | BTK | CD247 | -0.2196 | 0.5390 (0.4331-0.6708) | 0.0016 |
| P5 | C5 | CD96 | -0.1495 | 0.5374 (0.4318-0.6688) | 0.0013 |
| P6 | EGR2 | IL15 | -0.1344 | 0.4919 (0.3736-0.6476) | 0.0218 |
| P7 | LY9 | MAGEC2 | -0.1232 | 0.5243 (0.4109-0.6690) | 0.0106 |
| P8 | CXCL1 | IL1A | -0.1094 | 0.4930 (0.3918-0.6205) | <0.0001 |
| P9 | HLA-DPB1 | RRAD | -0.1075 | 0.3013 (0.1987-0.4567) | 0.0008 |
| P10 | CLEC4A | EOMES | -0.1030 | 0.3767 (0.2684-0.5285) | 0.0008 |
| P11 | EGR1 | NFKB1 | -0.0883 | 0.2786 (0.1913-0.4057) | <0.0001 |
| P12 | ITGA5 | MR1 | -0.0789 | 0.2138 (0.1534-0.2978) | <0.0001 |
| P13 | CCL11 | MARCO | -0.0405 | 0.4142 (0.3165-0.5419) | <0.0001 |
| P14 | ITGA1 | TLR4 | -0.0366 | 0.4327 (0.3338-0.5609) | <0.0001 |
| P15 | AXL | CEACAM1 | -0.0267 | 0.4631 (0.3714-0.5774) | <0.0001 |
| P16 | NUP107 | THY1 | 0.0071 | 1.8002 (1.4465-2.2404) | 0.0071 |
| P17 | SYT17 | THY1 | 0.0303 | 4.4975 (3.0313-6.6728) | <0.0001 |
| P18 | SAA1 | TGFB1 | 0.0664 | 2.3219 (1.7835-3.0229) | <0.0001 |
| P19 | AMMECR1L | MAP2K1 | 0.1327 | 3.1546 (2.1126-4.7105) | 0.0010 |
| P20 | CARD11 | IL1R1 | 0.1369 | 2.2173 (1.7767-2.7672) | <0.0001 |
| P21 | CD96 | CNOT4 | 0.1592 | 2.4168 (1.9148-3.0506) | <0.0001 |
| P22 | CD3G | DPP4 | 0.1845 | 2.2074 (1.7511-2.7825) | <0.0001 |
| P23 | CCL17 | PBK | 0.2113 | 2.6056 (1.8776-3.6159) | 0.0005 |
| P24 | AMMECR1L | IL4R | 0.2335 | 2.2186 (1.7213-2.8594) | <0.0001 |
| P25 | JAK2 | PDGFC | 0.2610 | 2.0628 (1.6211-2.6248) | 0.0002 |
| P26 | TLR4 | VEGFC | 0.3275 | 4.3226 (2.9333-6.3698) | <0.0001 |
| P27 | INPP5D | PVR | 0.3370 | 1.7815 (1.4169-2.2399) | 0.0395 |
| P28 | MEFV | SERPINB2 | 0.3974 | 2.3379 (1.8290-2.9883) | <0.0001 |
| P29 | ICAM2 | THY1 | 0.4698 | 6.1065 (3.8817-9.6066) | <0.0001 |

**Supplementary Table S4.** Area under the time-dependent ROC and C-index in different datasets.

| **Dataset** | **Number of patients** | **AUC** | | | **C-index (95% CI)** |
| --- | --- | --- | --- | --- | --- |
|  |  | **3-year** | **5-year** | **10-year** |  |
| **Meta-training dataset** | 724 | 0.85 | 0.85 | 0.79 | 0.79 (0.76-0.81) |
| **Meta-testing dataset** | 180 | 0.76 | 0.78 | 0.74 | 0.71 (0.66-0.77) |
| **Validation dataset 1** | 185 | 0.72 | 0.71 | 0.69 | 0.68 (0.62-0.75) |
| **Validation dataset 2** | 146 | 0.64 | 0.65 | 0.80 | 0.63 (0.57-0.70) |

**Supplementary Table S5.** Multivariate Cox proportional hazards regression of IRGPI combining other major clinicopathological features.

| **Dataset** | **Meta-training dataset** | |  | **Meta-testing dataset** | |  | **Validation dataset 1** | |  | **Validation dataset 2** | |
| --- | --- | --- | --- | --- | --- | --- | --- | --- | --- | --- | --- |
|  | **HR (95% CI) ^b^** | ***P* ^b^** |  | **HR (95% CI) ^b^** | ***P* ^b^** |  | **HR (95% CI) ^b^** | ***P* ^b^** |  | **HR (95% CI) ^b^** | ***P* ^b^** |
| IRGPI ^a^ | 3.21 (2.54-4.05) | <0.001 |  | 1.55 (1.06-2.28) | 0.025 |  | 1.81 (1.30-2.52) | <0.001 |  | 1.92 (1.38-2.68) | <0.001 |
| Age (per 10 years) | 1.15 (1.01-1.31) | 0.034 |  | 1.27 (0.99-1.62) | 0.060 |  | 1.54 (1.24-1.92) | <0.001 |  | 1.61 (1.24-2.07) | <0.001 |
| Gender |  |  |  |  |  |  |  |  |  | NA ^d^ | NA |
| Female | Ref. ^c^ | - |  | Ref. | - |  | Ref. | - |  | - | - |
| Male | 0.94 (0.71-1.24) | 0.658 |  | 1.31 (0.66-2.60) | 0.434 |  | 0.79 (0.45-1.39) | 0.410 |  | - | - |
| T stage |  |  |  |  |  |  |  |  |  | NA ^e^ | NA |
| Ta + T1 | Ref. | - |  | Ref. | - |  | Ref. | - |  | - | - |
| T2 + T3 + T4 | 3.35 (1.72-6.53) | <0.001 |  | 9.35 (2.72-32.20) | <0.001 |  | 1.47 (0.86-2.53) | 0.160 |  | - | - |
| Grade |  |  |  |  |  |  |  |  |  | NA | NA |
| Well/moderately differentiated | Ref. | - |  | Ref. | - |  | Ref. | - |  | - | - |
| Poorly differentiated | 1.06 (0.38-2.96) | 0.905 |  | 0.53 (0.05-5.06) | 0.578 |  | 0.77 (0.42-1.42) | 0.402 |  | - | - |
| ^a^ IRGPI: immune-related gene pair index. | | | | | | | | | | | |
| ^b^ Corresponding hazard ratio (HR) and *P* values of multivariate Cox regression model by combining all the available variables. | | | | | | | | | | | |
| ^c^ Reference group in Cox proportional hazards regression model with binary variables. | | | | | | | | | | | |
| ^d^ No records in at least one cohort. | | | | | | | | | | | |
| ^e^ Number of patients in reference is scarce with only three records for Ta + T1. | | | | | | | | | | | |

**Supplementary Table S6.** Restricted mean survival (RMS) time ratio between two risk groups in different datasets.

| **Cohorts** | **N_HRisk_** | **N_LRisk_** | **RMS_HRisk_ (95% CI) ^a^** | **RMS_LRisk_ (95% CI) ^a^** | **RMS ratio (95% CI) ^b^** | ***P*** |
| --- | --- | --- | --- | --- | --- | --- |
| Meta-training dataset | 362 | 362 | 54.47 (46.45-62.49) | 131.07 (114.91-147.22) | 0.42 (0.34-0.50) | < 0.001 |
| NMIBC ^c^ | 106 | 107 | 93.71 (88.09-99.32) | 103.64 (101.76-105.51) | 0.90 (0.85-0.96) | 0.002 |
| MIBC ^d^ | 255 | 256 | 41.30 (33.65-48.96) | 100.47 (86.65-114.29) | 0.41 (0.33-0.52) | < 0.001 |
| Meta-testing dataset | 90 | 90 | 53.45 (41.24-65.67) | 104.30 (92.70-115.91) | 0.51 (0.40-0.66) | < 0.001 |
| NMIBC | 30 | 31 | 85.31 (74.12-96.49) | 98.07 (98.07-98.07) | 0.87 (0.76-0.99) | 0.037 |
| MIBC | 59 | 60 | 40.18 (26.69-53.67) | 78.23 (61.81-94.65) | 0.51 (0.35-0.76) | 0.001 |
| Validation dataset 1 | 92 | 93 | 64.74 (51.47-78.01) | 96.98 (85.65-108.32) | 0.67 (0.53-0.85) | 0.001 |
| NMIBC | 54 | 54 | 80.40 (64.72-96.08) | 105.15 (91.46-118.85) | 0.77 (0.61-0.97) | 0.025 |
| MIBC | 38 | 39 | 47.91 (30.14-65.68) | 70.54 (52.56-88.52) | 0.68 (0.43-1.07) | 0.092 |
| Validation dataset 2 ^e^ | 73 | 73 | 47.00 (33.45-60.55) | 99.43 (76.30-122.56) | 0.47 (0.33-0.69) | < 0.001 |
| MIBC | 71 | 72 | 46.54 (32.91-60.16) | 97.52 (74.70-120.34) | 0.48 (0.33-0.69) | < 0.001 |
| ^a^ RMS time: months. | | | | | | |
| ^b^ RMS ratio = RMS_HRisk_/RMS_LRisk_. | | | | | | |
| ^c^ NMIBC: non-muscle invasive bladder cancer. | | | | | | |
| ^d^ MIBC: muscle invasive bladder cancer. | | | | | | |
| ^e^ Only three samples were considered as NMIBC (T1). | | | | | | |

**Supplementary Table S7.** Comparison of IRGPI with existing prognostic signatures for all-stage bladder cancer.

| **Signatures** | **HR (95% CI)** | ***P_1_* ^a^** | **C-index** | ***P_2_* ^b^** |
| --- | --- | --- | --- | --- |
| IRGPI (continous) | 2.50 (2.23-2.80) | < 0.001 | 0.73 | - |
| Cao et al. | 2.72 (2.13-3.46) | < 0.001 | 0.63 | < 0.001 |
|  |  |  |  |  |
| IRGPI (binary ^c^) |  | < 0.001 | 0.75 | - |
| LRisk | Ref. ^d^ |  |  |  |
| HRisk | 2.76 (2.28-3.34) |  |  |  |
| Mo et al. |  | 0.005 | 0.58 | < 0.001 |
| Differentiated | Ref. |  |  |  |
| Basal | 1.29 (1.08-1.55) |  |  |  |
| ^a^ P value calculated by univariate Cox proportional hazards regression model. | | | | |
| ^b^ P value calculated by C-index comparison with IRGPI as reference. | | | | |
| ^c^ Binary IRGPI risk group determined by cohort-specific median cutoff of IRGPI. | | | | |
| ^d^ Reference group in Cox proportional hazards regression model with binary variables. | | | | |

**Supplementary Table S8.** Comparison of IRGPI with existing prognostic signatures for MIBC-specific bladder cancer.

| **Cohort ^a^** | **IRGPI** | | |  | **Abudurexiti et al. ^b^** | | |  | **Goux et al. ^c^** | | |
| --- | --- | --- | --- | --- | --- | --- | --- | --- | --- | --- | --- |
|  | **HR (95% CI)** | ***P*** | **C-index** |  | **HR (95% CI)** | ***P*** | **C-index (*P* ^d^)** |  | **HR (95% CI)** | ***P*** | **C-index (*P* ^d^)** |
| TCGA-BLCA  (MIBC n = 316) | 3.89  (2.88-5.25) | < 0.001 | 0.70 |  | 2.72  (2.16-3.41) | < 0.001 | 0.70  (ns) |  | 2.72  (1.06-7.00) | 0.038 | 0.55  (<0.001) |
| GSE13507  (MIBC n = 62) | 1.95  (1.05-3.63) | 0.036 | 0.63 |  | 2.72  (1.81-4.08) | < 0.001 | 0.76  (0.006) |  | 2.72  (0.17-43.50) | 0.480 | 0.56  (ns) |
| ^a^ Samples within the cohorts used here are those with confirmed muscle-invasiveness status as muscle-invasive bladder cancer (MIBC). | | | | | | | | | | | |
| ^b^ Abudurexiti et al. is a 12-gene prognostic signature. | | | | | | | | | | | |
| ^c^ Goux et al. is a 3-gene prognostic signature. | | | | | | | | | | | |
| ^d^ Comparison for C-index with IRGPI and ns indicates no significance (*P* > 0.05). | | | | | | | | | | | |

**SUPPLEMENTARY FIGURES**


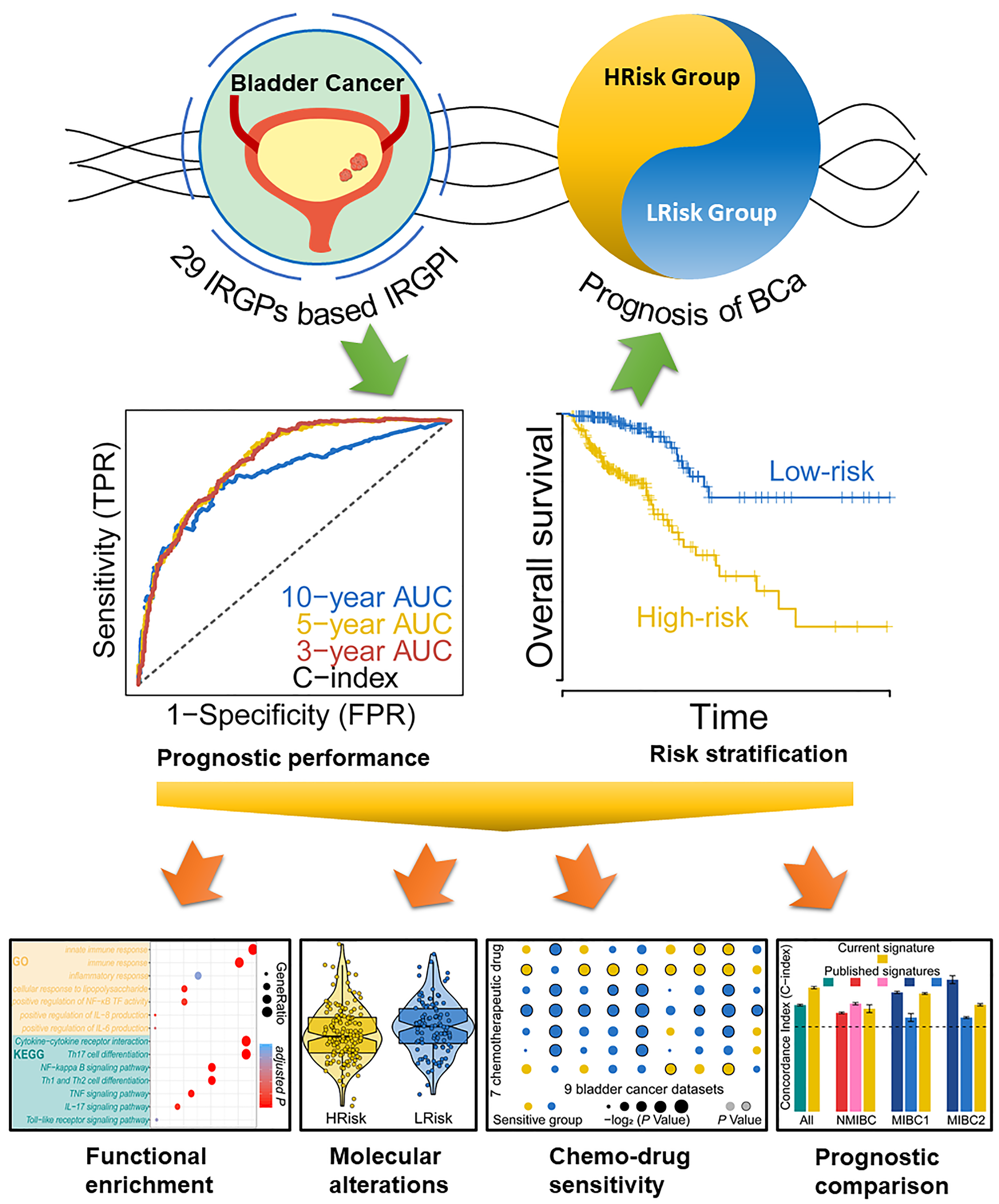


**Figure S1. Flow chart of the study.** A total of 445 prognostic IRGPs was first derived and was further applied to multivariate Cox proportional hazards regression with adaLASSO penalty, resulting in an IRGPI that consisted of 29 IRGPs, including 51 IRGs. The prognostic value of the IRPGI was validated by C-index, time-dependent ROC at 3-year, 5-year and 10-year, and Kaplan-Meier estimator in meta-training, meta-testing, and two independent external validation datasets. To gain deep biological understanding of IRGPI, we performed GO and KEGG enrichment analyses and discovered functional pathways that these IRGs were significantly enriched in. We stratified two risk group (*i.e.*, HRisk and LRisk groups) according to cohort-specific median cutoff of IRGPI, and examined the molecular alteration (*e.g.*, somatic mutation and consensus molecular subtypes) and differentiated chemosensitivity in different risk groups. The performance of IRGPI in predicting prognosis of BCa was further compared with other public prognostic signatures, including four stage-specific signatures.

**
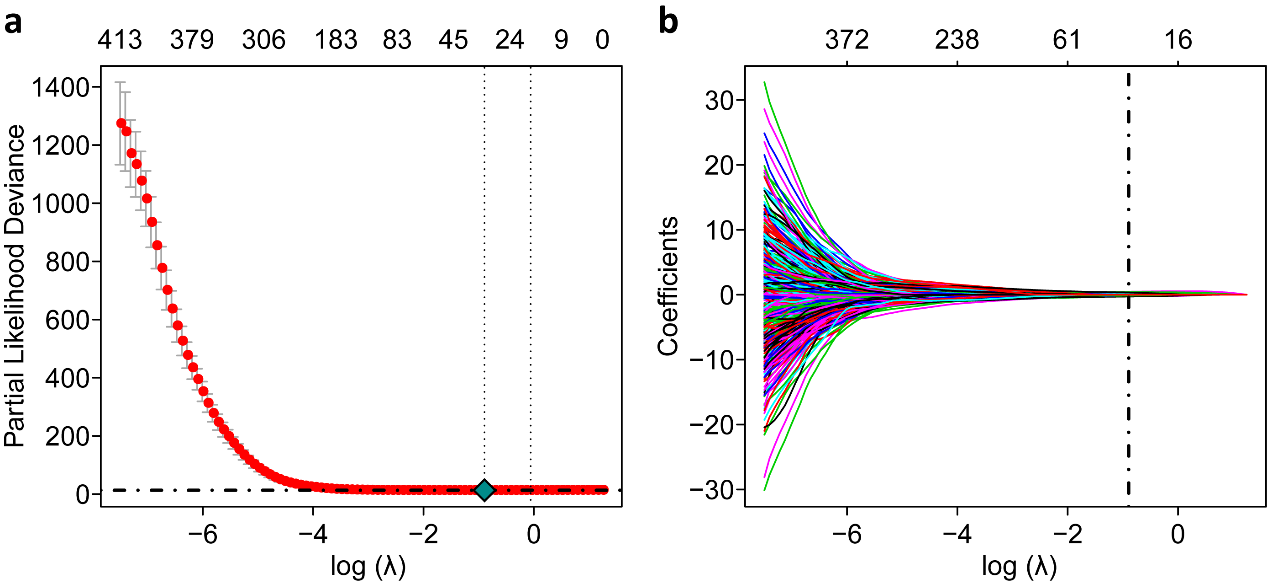
**

**Figure S2.** **Feature selection using multivariate Cox proportional hazards regression model with adaptive LASSO (adaLASSO) penalty.** a) Tuning parameter λ selection in the adaLASSO model used 10-fold cross-validation by minimum criteria. The partial likelihood deviance was plotted vs. log(λ). Dotted vertical lines were drawn at the optimal values by using the minimum criteria. A λ of 0.409 with log(λ) of −0.893 was chosen according to 10-fold cross-validation. b) addLASSO coefﬁcient proﬁles of the 445 immune-gene pairs. A coefﬁcient proﬁle plot was produced against the log(λ) sequence. A vertical line was drawn at the value selected by 10-fold cross-validation, where the optimal λ resulted in 29 non-zero coefﬁcients.


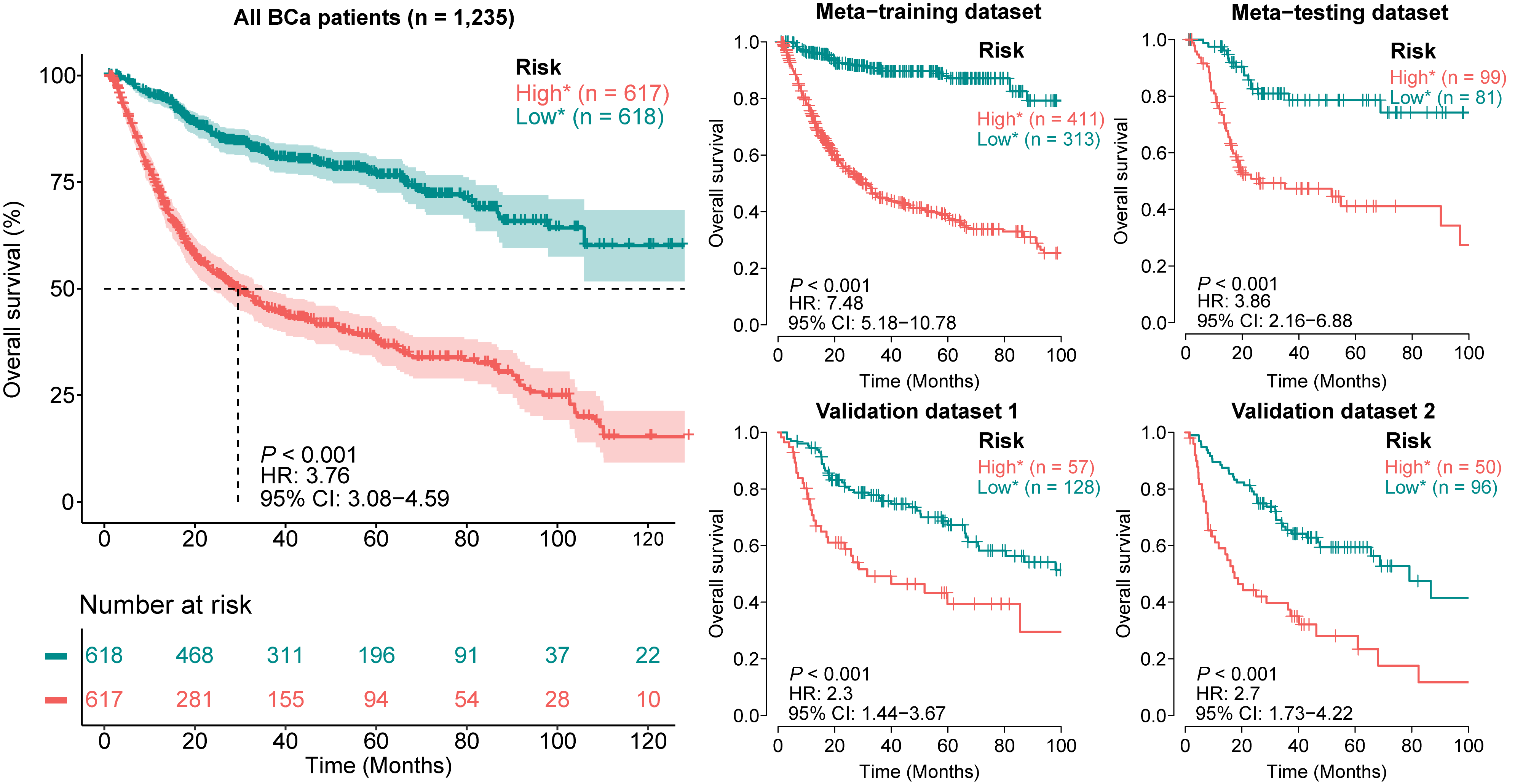


**Figure S3. Universal prognostic value of IRGPI.** A general cutoff of 1.195 was calculated using the median value of IRGPI among 1,235 BCa patients. Using such cutoff, the entire 1,235 samples and four datasets (*i.e.*, meta-training, meta-testing, validation 1 and validation 2 datasets) were re-separated into HRisk* and LRisk* groups. Consistently, Kaplan-Meier curves showed that HRisk* groups presented with significantly poor overall survival than matched LRisk* groups (all, log-rank test *P* < 0.001). Hazard ratio (HR) and 95% confidence intervals (95% CI) were estimated by Cox proportional hazards regression.


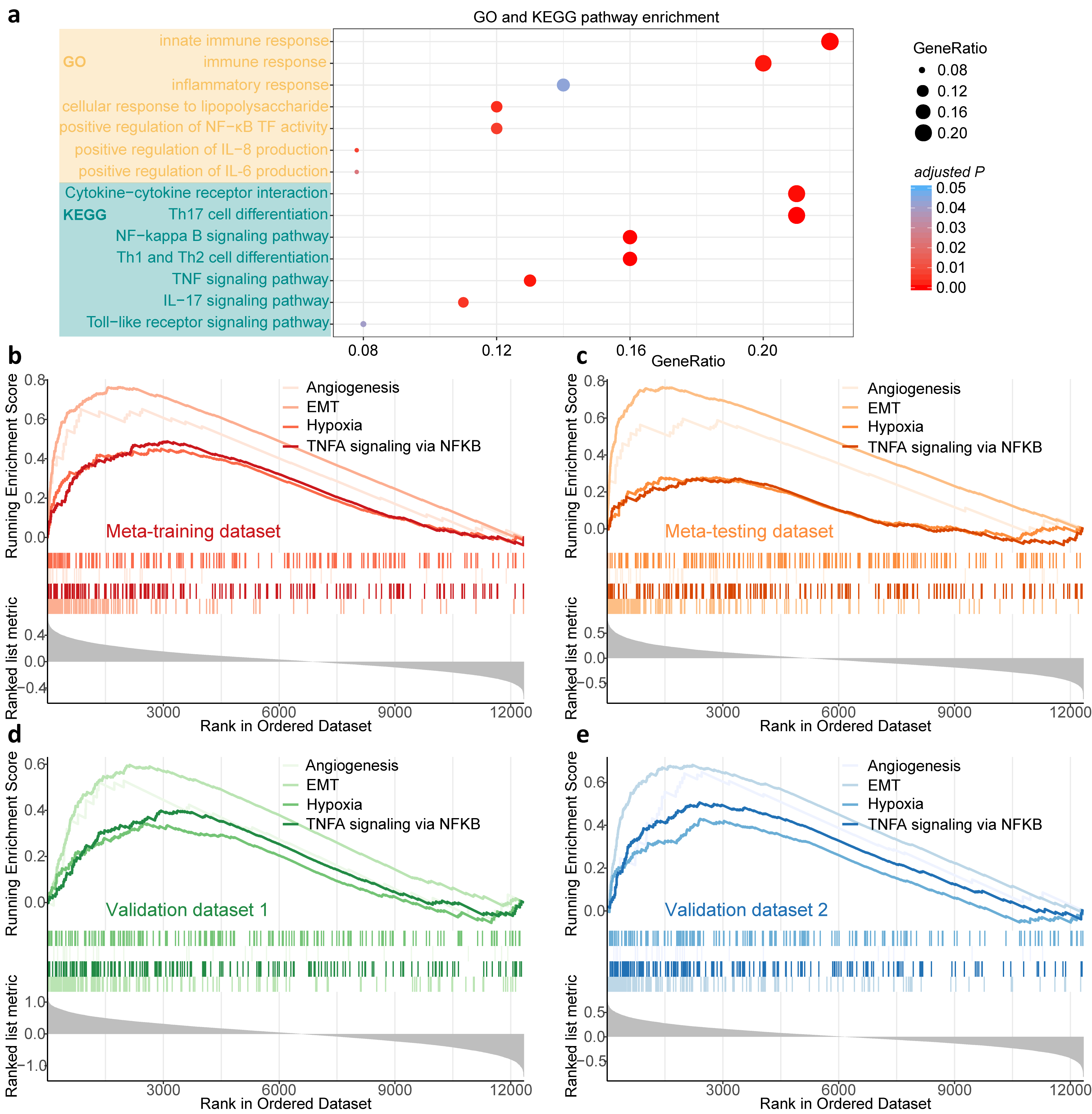


**Figure S4. Association between IRGPI and biological dysfunction.** a) Dotplot showing the pathways enrichment of 51 IRGP-associated genes by GO and KEGG analyses. GSEA revealed that HRisk groups in b) meta-training, c) meta-testing, d) validation 1 and e) validation 2 datasets were enriched for poor survival and immune suppression-related pathways, including tumor necrosis factor-α (TNFA), epithelial-mesenchymal transition (EMT), angiogenesis and hypoxia.


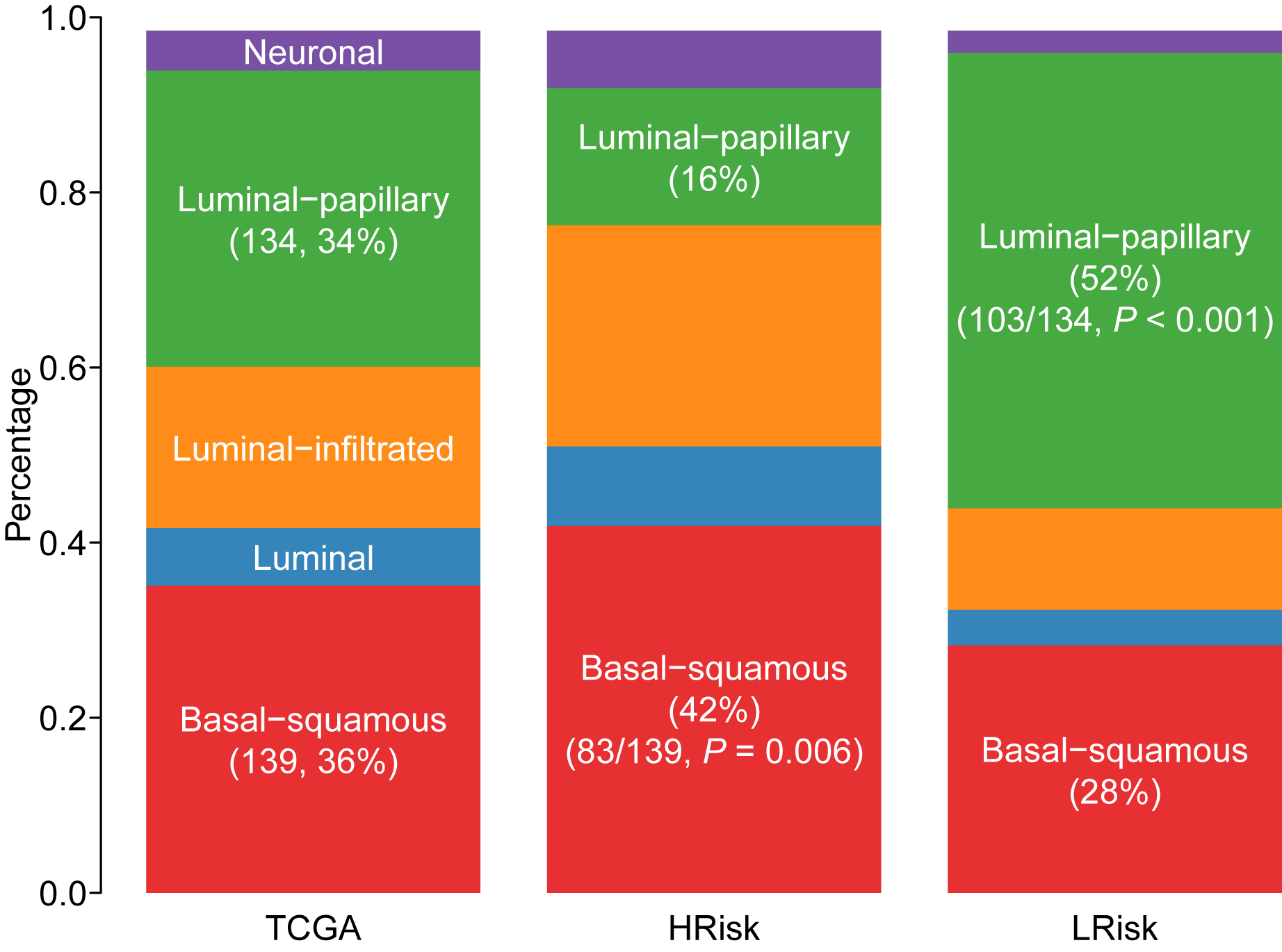


**Figure S5. Association between TCGA defined molecular subtype and IRGPI stratified risk group.** Molecular classification of bladder cancer defined from TCGA includes basal-squamous (139, 36%), luminal (26, 7%), luminal-infiltrated (73, 19%), luminal-papillary (134, 34%), and neuronal (18, 5%). Barplot showing that HRisk group in TCGA cohort enriched in basal-squamous subtype (83 out of 139, 60%; *P* = 0.006) while LRisk group enriched in luminal-papillary subtype (103 out of 134, 77%; *P* < 0.001).

**
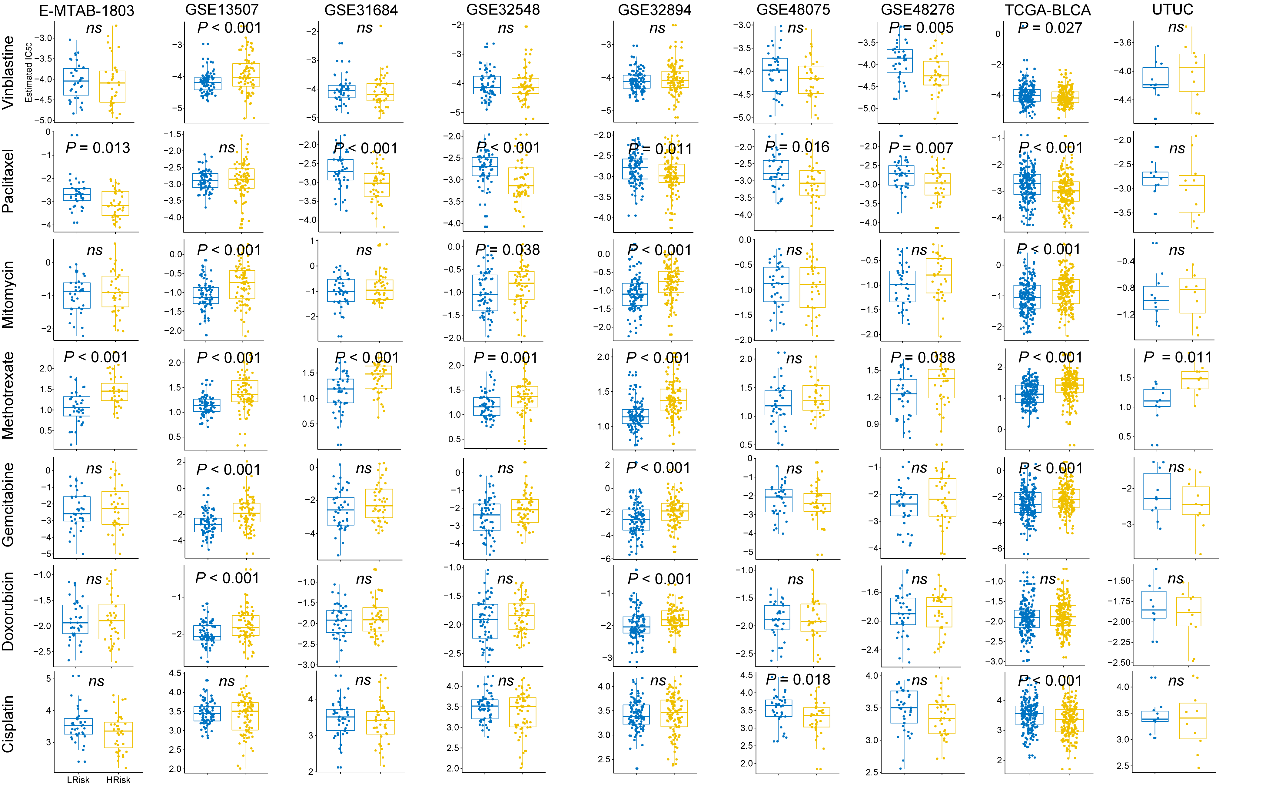
**

**Figure S6. Differential putative chemotherapeutic response.** The box plots of the estimated IC_50_ of HRisk (yellow box) and LRisk (blue box) group for seven chemotherapeutic drugs are shown, including cisplatin, paclitaxel, gemcitabine, methotrexate, vinblastine, doxorubicin and mitomycin. Risk groups for nine cohorts used in this study were determined by cohort-specific median cutoff of IRGPI. Statistical *P* values are calculated by two-sample Mann-Whitney test.


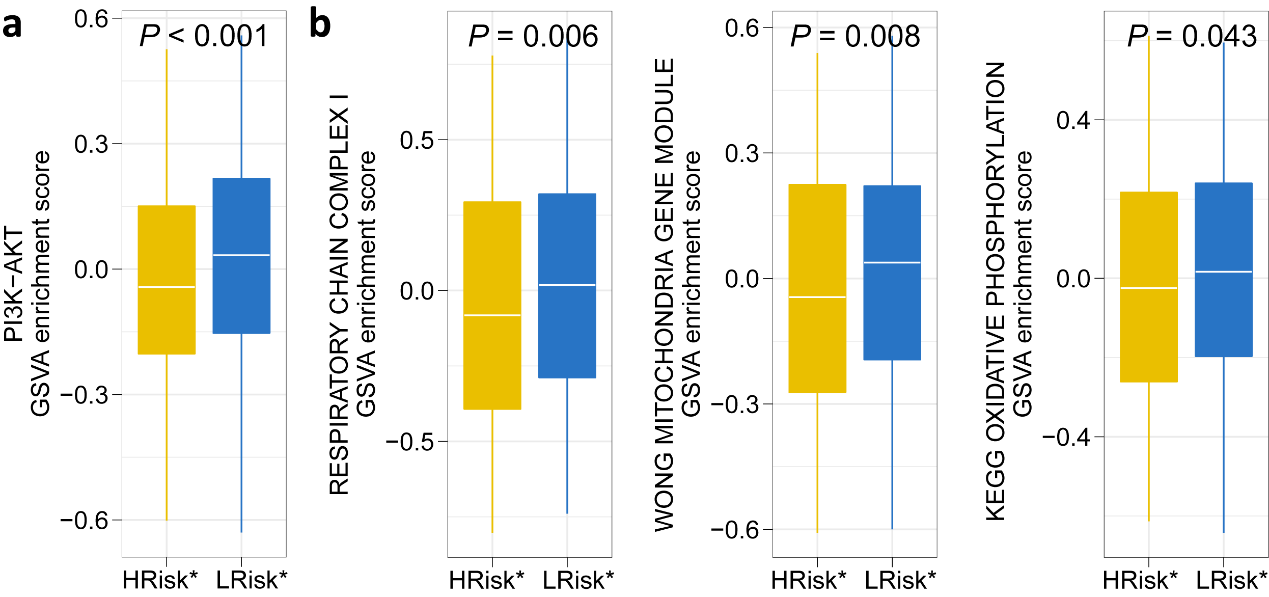


**Figure S7. Association between pathway dysfunction and differential drug sensitivity.** a) Boxplot showing PI3K-AKT signaling was activated in *FGFR3*-enriched LRisk* group (defined by general cutoff of 1.195) cells and may result in resistance of cisplatin. b) Boxplots showing highly activated complex I/mitochondrial complex in LRisk* group may converge to low recurrence rate of mitomycin-C chemotherapy.


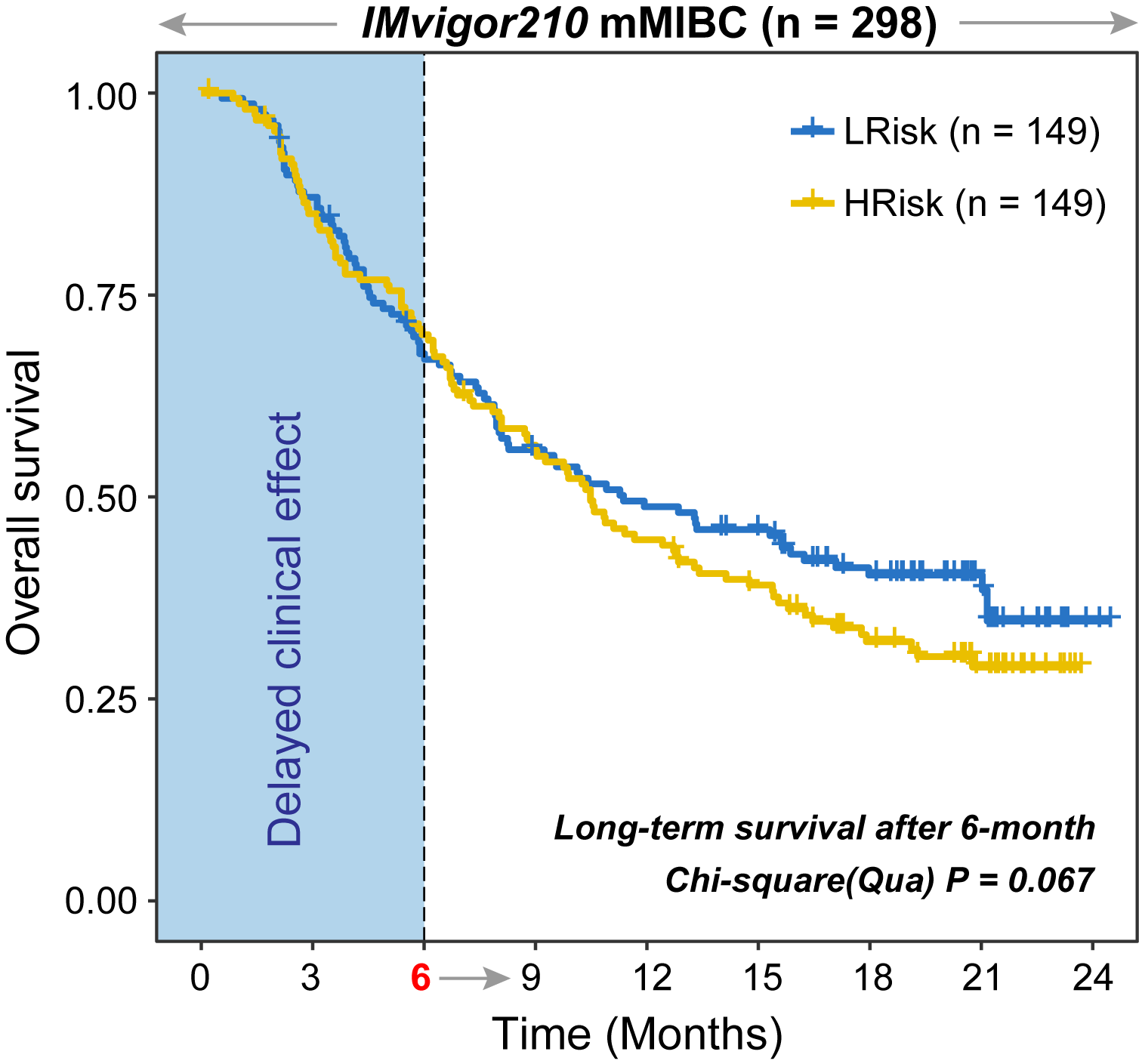


**Figure S8. Overall survival Kaplan-Meier curves of two risk groups in IMvigor210 metastatic MIBC cohort.** Given the delated clinical effect of immunotherapy, the association between the risk group and PD-L1 blockade response was reflected by long-term survival analysis after 6-month of treatment using non-proportional hazards statistical approach (*i.e.*, Chi-square [Quadratic test]). Patients were stratified by the median cutoff of IRGPI.

**REFERENCES**

1. Su X, Lu X, Bazai SK et al. Comprehensive integrative profiling of upper tract urothelial carcinomas, Genome Biology 2021;22:7.

2. Wagner GP, Kin K, Lynch VJ. Measurement of mRNA abundance using RNA-seq data: RPKM measure is inconsistent among samples, Theory in biosciences 2012;131:281-285.

3. Lazar C, Meganck S, Taminau J et al. Batch effect removal methods for microarray gene expression data integration: a survey, Briefings in Bioinformatics 2012;14:469-490.

4. Li B, Cui Y, Diehn M et al. Development and validation of an individualized immune prognostic signature in early-stage nonsquamous non–small cell lung cancer, JAMA oncology 2017;3:1529-1537.

5. Nie H, Bu F, Xu J et al. 29 immune-related genes pairs signature predict the prognosis of cervical cancer patients, Scientific reports 2020;10:14152.

6. Wang S, Xu X. An Immune-Related Gene Pairs Signature for Predicting Survival in Glioblastoma, Frontiers in oncology 2021;11.

7. Zhang L, Zhu P, Tong Y et al. An immune-related gene pairs signature predicts overall survival in serous ovarian carcinoma, OncoTargets and Therapy 2019;12:7005-7014.

8. Meng L, He X, Zhang X et al. Predicting the clinical outcome of melanoma using an immune-related gene pairs signature 2020;15:e0240331.

9. Cesano A. nCounter® PanCancer immune profiling panel (NanoString technologies, Inc., Seattle, WA), Journal for immunotherapy of cancer 2015;3:42.

10. Kim S, Lin C-W, Tseng GC. MetaKTSP: a meta-analytic top scoring pair method for robust cross-study validation of omics prediction analysis, Bioinformatics 2016;32:1966-1973.

11. Zou H. The adaptive lasso and its oracle properties, Journal of the American Statistical Association 2006;101:1418-1429.

12. Yu G, Wang L-G, Han Y et al. clusterProfiler: an R package for comparing biological themes among gene clusters, Omics: a journal of integrative biology 2012;16:284-287.

13. Subramanian A, Tamayo P, Mootha VK et al. Gene set enrichment analysis: a knowledge-based approach for interpreting genome-wide expression profiles, Proceedings of the National Academy of Sciences 2005;102:15545-15550.

14. Ritchie ME, Phipson B, Wu D et al. limma powers differential expression analyses for RNA-sequencing and microarray studies, Nucleic Acids Research 2015;43:e47-e47.

15. Li B, Cui Y, Nambiar DK et al. The immune subtypes and landscape of squamous cell carcinoma, Clinical cancer research 2019;25:3528-3537.

16. Catasus L, D'Angelo E, Pons C et al. Expression profiling of 22 genes involved in the PI3K–AKT pathway identifies two subgroups of high-grade endometrial carcinomas with different molecular alterations, Modern Pathology 2010;23:694-702.

17. Hänzelmann S, Castelo R, Guinney J. GSVA: gene set variation analysis for microarray and RNA-seq data, BMC bioinformatics 2013;14:1-15.

18. Mayakonda A, Lin D-C, Assenov Y et al. Maftools: efficient and comprehensive analysis of somatic variants in cancer, Genome research 2018;28:1747-1756.

19. Bailey MH, Tokheim C, Porta-Pardo E et al. Comprehensive characterization of cancer driver genes and mutations, Cell 2018;173:371-385. e318.

20. Kamoun A, de Reynies A, Allory Y et al. A consensus molecular classification of muscle-invasive bladder cancer, European urology 2019.

21. Geeleher P, Cox NJ, Huang RS. Clinical drug response can be predicted using baseline gene expression levels and in vitro drug sensitivity in cell lines, Genome Biology 2014;15:R47.

22. Jiang P, Gu S, Pan D et al. Signatures of T cell dysfunction and exclusion predict cancer immunotherapy response, Nature medicine 2018;24:1550-1558.

23. Ghandi M, Huang FW, Jané-Valbuena J et al. Next-generation characterization of the cancer cell line encyclopedia, Nature 2019;569:503-508.

24. Oresta B, Pozzi C, Braga D et al. Mitochondrial metabolic reprogramming controls the induction of immunogenic cell death and efficacy of chemotherapy in bladder cancer, Science Translational Medicine 2021;13.

25. Mariathasan S, Turley SJ, Nickles D et al. TGFβ attenuates tumour response to PD-L1 blockade by contributing to exclusion of T cells, Nature 2018;554:544-548.

26. Subudhi SK, Vence L, Zhao H et al. Neoantigen responses, immune correlates, and favorable outcomes after ipilimumab treatment of patients with prostate cancer, Science Translational Medicine 2020;12.

27. Cao R, Yuan L, Ma B et al. An EMT‐related gene signature for the prognosis of human bladder cancer, Journal of Cellular and Molecular Medicine 2019.

28. Mo Q, Nikolos F, Chen F et al. Prognostic power of a tumor differentiation gene signature for bladder urothelial carcinomas, JNCI: Journal of the National Cancer Institute 2018;110:448-459.

29. van der Heijden AG, Mengual L, Lozano JJ et al. A five-gene expression signature to predict progression in T1G3 bladder cancer, European Journal of Cancer 2016;64:127-136.

30. Dyrskjøt L, Reinert T, Novoradovsky A et al. Analysis of molecular intra-patient variation and delineation of a prognostic 12-gene signature in non-muscle invasive bladder cancer; technology transfer from microarrays to PCR, British journal of cancer 2012;107:1392.

31. Le Goux C, Vacher S, Pignot G et al. mRNA Expression levels of genes involved in antitumor immunity: Identification of a 3-gene signature associated with prognosis of muscle-invasive bladder cancer, Oncoimmunology 2017;6:e1358330.

32. Abudurexiti M, Huyang X, Zhongwei J et al. Development and external validation of a novel 12-gene signature for prediction of overall survival in muscle-invasive bladder cancer, Frontiers in oncology 2019;9:856.

33. Qiu H, Hu X, He C et al. Identification and validation of an individualized prognostic signature of bladder cancer based on seven immune related genes, Frontiers in genetics 2020;11:12.

34. Luo Y, Chen L, Zhou Q et al. Identification of a prognostic gene signature based on an immunogenomic landscape analysis of bladder cancer, Journal of cellular and molecular medicine 2020;24:13370-13382.

35. Jiang W, Zhu D, Wang C et al. An immune relevant signature for predicting prognoses and immunotherapeutic responses in patients with muscle‐invasive bladder cancer (MIBC), Cancer medicine 2020;9:2774-2790.

36. Xu N, Ke Z-B, Lin X-D et al. Development and validation of a molecular prognostic index of bladder cancer based on immunogenomic landscape analysis, Cancer Cell International 2020;20:1-14.

37. Liang F, Zhang S, Wang Q et al. Treatment effects measured by restricted mean survival time in trials of immune checkpoint inhibitors for cancer, Annals of Oncology 2018;29:1320-1324.

38. Logan BR, Klein JP, Zhang MJ. Comparing treatments in the presence of crossing survival curves: an application to bone marrow transplantation, Biometrics 2008;64:733-740.
